# Anticandidal applications of selenium nanoparticles biosynthesized with *Limosilactobacillus fermentum* (OR553490)

**DOI:** 10.1186/s11671-024-04055-z

**Published:** 2024-07-09

**Authors:** Esraa Ali Mohamed, Mohamed Marzouk El‑Zahed

**Affiliations:** Department of Botany and Microbiology, Faculty of Science, Damietta University, New Damietta, 34517 Egypt

**Keywords:** *Limosilactobacillus fermentum*, Biosynthesis, Optimization, Characterization, Anticandidal, *Candida albicans*

## Abstract

*Candida albicans* is one of the most dangerous pathogenic fungi in the world, according to the classification of the World Health Organization, due to the continued development of its resistance to currently available anticandidal agents. To overcome this problem, the current work provided a simple, one-step, cost-effective, and safe technique for the biosynthesis of new functionalized anticandidal selenium nanoparticles (Se NPs) against *C. albicans* ATCC10231 using the cell-free supernatant of *Limosilactobacillus fermentum* (OR553490) strain. The bacterial strain was isolated from yogurt samples available in supermarkets, in Damietta, Egypt. The mixing ratio of 1:9 v/v% between cell-free bacterial metabolites and sodium selenite (5 mM) for 72 h at 37 °C were the optimum conditions for Se NPs biosynthesis. Ultraviolet–visible spectroscopy (UV–Vis), Fourier transform infrared spectroscopy (FT-IR), transmission electron microscopy (TEM), X-ray diffraction (XRD), Zeta analyses, and elemental analysis system (EDS) were used to evaluate the optimized Se NPs. The Se NPs absorption peak appeared at 254 nm. Physicochemical analysis of Se NPs revealed the crystalline-shaped and well-dispersed formation of NPs with an average particle size of 17–30 nm. Se NPs have − 11.8 mV, as seen by the zeta potential graph. FT-IR spectrum displayed bands of symmetric and asymmetric amines at 3279.36 cm^−1^ and 2928.38 cm^−1^, aromatic and aliphatic (C–N) at 1393.32 cm^−1^ and 1237.11.37 cm^−1^ confirming the presence of proteins as stabilizing and capping agents. Se NPs acted as a superior inhibitor of *C. albicans* with an inhibition zone of 26 ± 0.03 mm and MIC value of 15 µg/mL compared to one of the traditional anticandidal agent, miconazole, which revealed 18 ± 0.14 mm and 75 µg/mL. The cytotoxicity test shows that Se NPs have a low toxic effect on the normal keratinocyte (IC_50_ ≈ 41.5 μg/mL). The results indicate that this green synthesis of Se NPs may have a promising potential to provide a new strategy for drug therapy.

## Background

The genus *Candida* comprises over 200 species of which 15 have been isolated from infections in humans and animals. The most prevalent pathogens are *Candida albicans*, *C. glabrata, C. parapsilosis*, *C. tropicalis*, and *C. krusei* [1]. *C. albicans* as an opportunistic fungal pathogen is a typical component of the microbiota of the human digestive tract. Even though there are only 200 species in the genus *Candida*, it can cause up to 75% of candidal infections. On the other hand, bacterial normal flora acquired host defense mechanisms to allow *Candida* organisms to proliferate and endure as commensals. Nonetheless, even a small alteration to the host’s biological milieu or defensive mechanism can let *C. albicans* develop into a pathogen that can cause potentially fatal infections [2]. The microbiota in healthy hosts often consists of a balanced mix of energy and metabolites. Potentially harmful germs are kept from growing out of control by the homeostatic balance. Dysbiosis, or the imbalance of the microbial community, is frequently linked to illnesses in humans [3]. While some oral bacteria promote *C. albicans* biofilm production, others hinder it. The expression of *C. albicans* virulence genes, as well as the growth of C. albicans hyphae and biofilm, were found to be up regulated by *Streptococcus* and *Actinomyces* species in an in vitro measurement of mixed species biofilm generated on the salivary pellicle. Additionally, *S. oralis* promoted the growth of *C. albicans* hyphae and biofilms [4]. *C. albicans* is the most dangerous *Candida* species according to the World Health Organization (WHO) fungal priority pathogens list [5]. Even though *C. albicans* is a typical microbiome, it can foster opportunistic infections that might endanger both human and animal lives. *C. albicans* typically affects the whole part of the body from the skin to the lower respiratory tract, oropharynx, gastrointestinal tract, and genitourinary system [6]. The term “candidiasis” refers to a group of illnesses that vary from severe infections to less significant, while invasive candidiasis (IC) includes serious conditions such as candidemia, endocarditis, disseminated infections, infections of the central nervous system, endophthalmitis, and osteomyelitis. Also, candidiasis risk factors include the use of corticosteroids, invasive procedures, and harsh chemotherapy [7]. Around 70% of fungal infections worldwide are brought on by *C. albicans*. Throughout the past few decades, it has consistently been the main contributor to invasive infections that can be fatal [8]. *C. albicans* can be treated through chemical anticandidal drugs like the Azole group which are two subgroups: imidazole and triazole subgroup, but these drugs have highly severe side effects such as hypertriglyceridemia, elevated liver enzymes, rash, pedal edema, hepatotoxicity [9]. The *Candida* sp. virulence increases due to its ability to resist the available antifungal drugs such as the azole group that occur due to modifications of the target enzyme or due to reduced access of the drug to the target [1, 5, 9].

Nanotechnology was a recent science field which studied matters at the nanoscale dimension and changes their properties. NPs are 1–100 nm in size and have advantages of chemical stability, potential antifungal effects, low toxicity, and low pathogen resistance [10, 11]. NPs such as silver, copper, gold, iron oxide, and selenium have critical roles in agriculture, food, the environment, and the nanomedicine field [12, 13]. Potential NPs could disrupt the cell membrane of the microbial cells through a mechanism of inhibiting the activity of the enzyme Lanosterol 14-α-demethylase, which is involved in the cholesterol analogue (ergosterol) biosynthesis and the largest sterol element of the fungal cell membrane. Also, NPs might induce reactive oxygen species (ROS), inhibition of spore germination, and protein regulation [14–16]. Among different NPs, selenium NPs (Se NPs) were preferred due to several advantages such as their essential role in improving human health, via seleno-proteins, antioxidant defense, cell signaling, immunological modulation, and other metabolic activities [17]. In addition, Se NPs display several benefits, such as low toxicity, high bioavailability, and degradability so, it’s safe for clinical administration and outstanding in nanomedicine as anticancer, antiviral activity, and antimicrobial [18, 19]. Also, Se is used in several pathophysiological diseases like cardiovascular diseases, cancer, diabetes, neurodegenerative diseases, and so on, due to its activity as an antioxidant and anti-inflammatory [20]. Through the previous studies, the biological methods for Se NPs synthesis were limitless. Thus, using microorganisms might gain great attention as new bio-nano factories that convert the ion metals into metal NPs through biotransformation [21].

These NPs can be synthesized by chemical, physical, and biological methods, but the chemical and physical methods are not preferred due to high thermal conditions, hazardous chemicals, acidic pH, and highly toxic and unsafe methods than the biological method [22]. The biological synthesis of NPs is preferable due to its low cost, simplicity, safety, increased biocompatibility and stability, and non-toxic, high-productivity method for the production of NPs [23]. The biological method depends on the green chemistry that can be performed by living organisms or their natural secondary metabolites such as proteins including plants [24, 25], fungi [26], yeast [27], algae [28], non-living viruses [29] and bacteria [23, 30, 31]. Among the significant categories of microorganisms involved in biosynthesis of NPs, the probiotic lactic acid bacteria (LAB) are recommended as a safe, rapid, easy to culture, available in dairy products and cheap bio-nano-factory for NPs production [32, 33]. They can be facultative anaerobes, even though they are sometimes classified as aerotolerant anaerobes. LABS, which are extensively dispersed in nature, is the main microflora of milk and its derivatives. LAB produce different antimicrobial substances such as formic acid, hydrogen peroxide, ethanol, acetone, and bacteriocins that might show synergistic action with NPs [34]. These compounds endow them with the ability to preserve food, acting as a natural means of competition to outcompete other microorganisms occupying the same niche [35]. Moreover, LAB was reported to produce a high yield of secondary metabolites and nourish with benefit proteins [36]. Different LAB strains were reported to biosynthsize NPs with varying sizes, ranging from 50 to 100 nm (*S. thermofilus*), 100–200 nm (*Lactobacillus* sp.), and 400–500 nm (*Bifidobacter* sp.) [32, 35]. They pay close attention to nutrition. These bacteria get their energy from substrate-level phosphorylation. The current study aimed to use *Limosilactobacillus fermentum* OR553490 to bio-reduce Se salt into selenium NPs (Se NPs) extracellularly, characterize Se NPs, and in vitro test their potential as an anticandidal agent. In addition, application of the biosynthesized Se NPs in different medical products such as topical cream and shampoo as newly combinations with chitosan (CS), alginate (Alg) and panthenol to enhance their antimicrobial action.

## Materials and methods

### Materials

Sodium selenite (Na_2_SeO_3_) and miconazole were purchased from Sigma Aldrich, St. Louis, MO, USA. *Candida albicans* ATCC10231 strain was used in the anticandidal activity investigations. Culture media was purchased from Difco Laboratories, Detroit, Mich. Chemicals were purchased from Oxoid Ltd., England.

### Sample collection

Zabady, as previously mentioned by Altalhi et al. [37], is an Arabian yogurt produced from a 1:1 ratio of buffalo to cow milk. Samples of various Egyptian zabady (a total of 37 samples) were gathered in October and September from several supermarkets in the northern Egyptian cities of Damietta Governorate, which includes New Damietta, Kafr Saad, and Ras Elbar. Every sample was collected in sterile cups, brought to the Microbiology Laboratory, Faculty of Science, Damietta University, in an ice box, and kept in a refrigerator.

### Isolation and purification of LAB *bacteria*

DE Man, Rogosa, Sharpe (MRS) agar medium was prepared and sterilized using an autoclave (121 °C, 1.5 atm). After sterilization, leave the media to cool down to (42–45 °C). Serial dilutions from different zabady samples were prepared (10^–1^ to 10^–6^). About 0.1 mL of each dilution was added into a sterile Petri dish and the autoclaved culture media were poured under aseptic conditions. The inoculated agar plates were mixed well and incubated at 37 °C for 24 h under anaerobic conditions. After incubation time, a single colony from each different bacterial colony was transferred into new agar plates [38].

### Phenotypic characterization

After the purification steps, each single colony was stained using Gram stain followed by microscopic examination, morphological characterization, and testing of the catalase and oxidase activity. Only Gram-positive and catalase-negative bacterial isolates were selected, purified, and preserved on agar slants for further use [39].

### Screening for Se NPs production

A 0.5 McFarland standard (1 × 10^6^ cell/mL) from each different bacterial isolate was prepared in 100 mL MRS broth flasks for 24 h at 37 °C. The inoculated flasks were incubated overnight at 150 rpm and 37 °C. After the incubation period, bacterial cells were collected using centrifugation at 5000 rpm for 15 min while bacterial metabolites were filtered using a 0.22 μm syringe filter (Millex GV, Millipore) and transferred into another clean flask. 1 mM from Na_2_SeO_3_ solution was prepared and added into the flasks of bacterial metabolite by the ratio 1:1 (v/v), then incubated in a shaking incubator at 150 rpm at 37 °C until the color changed from colorless to red color. The produced Se NPs using different bacterial isolates were collected using centrifugation at 30,000 rpm for 20 min and lyophilized after being rinsed four times with distilled water [38]. All produced Se NPs reaction mixtures were measured spectrophotometrically to detect the Se NPs biosynthesis using double beam spectrum UV–Vis spectrophotometer V-760 (JASCO, UK).

### Evaluation of anticandidal action of the biosynthesized Se NPs using LAB isolates

Aliquot 150 μg/mL of different produced Se NPs from each LAB isolate was prepared and tested as an anticandidal agent in comparison to the standard anticandidal; miconazole according to the guidelines of the Clinical and Laboratory Standards Institute (CLSI) [40]. A 1 × 10^6^ cell/mL of *C. albicans* ATCC10231 Sabouraud dextrose agar (SDA) plates were prepared and used during the agar well diffusion method. After incubation at 28 °C for 24 h, zones of inhibition (ZOI) were measured in mm to determine the highest anticandidal Se NPs.

### Molecular diagnosis of the selected LAB isolates

The selected lactobacillus isolate genotypic identification was done at the Animal Health Research Institute, Giza (Egypt). Bacterial cells of the isolate were collected from overnight bacterial growth on MRS broth medium using centrifugation at 5000 rpm for 15 min, washed 3 times TE buffer (10 mM tris chloride, 1 mM EDTA, pH8.0), and resuspended in 350 μL TE buffer and 20 mg/mL of lysozyme (Sigma, USA). Tubes were vortexed, 350 μl of 10% SDS, and 100 µg/mL proteinase-K (Vivantis Technologies, Malaysia) were added, along with 100 g per mL, and then incubated at 37 °C for 1 h. After centrifugation, 200 μl ethanol (96%) was added, vortexed, centrifugated, and then applied to a QIAamp mini spin column (QIAamp DNA Mini Kit, catalog no.51304). After centrifugation at 8000 rpm for 1 min, the QIAamp mini spin column was transferred into a new collection tube, and the filtrate was discarded. The QIAamp mini spin column was washed several times using a buffer. 100 μL 1/10 TE buffer was added and stored at – 20 °C until used for DNA sequencing [41].

The genomic DNA was detected using electrophoresis on 1% agarose gel electrophoresis prepared in TAE buffer (0.04 M Tris-acetate and 0.001 M EDTA, pH 8.0) and ethidium bromide (10 mg/mL). Then, the agarose gel was examined using a UV transilluminator. A Gel Pilot 100 bp plus ladder (cat. no. 239045, QIAGEN, USA) was used as a molecular mass marker.

Amplification was done using oligonucleotide primers sequences (Metabion, Germany) including F27 (5' AGAGTTTGATCMTGGCTCAG 3') and R1492 (5' TACGGYTACCTTGTTACGACTT 3') to identify the selected isolate. The initial denaturation was done at 94°C for 15 min followed by 35 cycles of denaturation at 94 °C for 30 s, annealing at 56°C for 1 min, and extension at 72°C for 1 min [42].

The PCR product was sequenced in forward and reverse directions using Applied Biosystems 3130 automated DNA Sequencer (ABI, 3130, USA), a ready reaction Bigdye Terminator V3.1 cycle sequencing kit (Perkin-Elmer/Applied Biosystems, No. 4336817, Foster City, CA).

A BLAST^®^ analysis (Basic Local Alignment Search Tool) was performed to match the best similarities with other related sequences and establish sequence identity to GenBank accessions [43]. Phylogenetic analysis was performed using the CLUSTAL W multiple sequence alignment program, version 12.1 of MegAlign module of Lasergene DNAStar software Pairwise (Madison, Wisconsin, USA) [44].

The neighbor-joining was performed using the maximum composite likelihood methods and the phylogenetic tree analyses were viewed and analyzed using MEGA6. The bootstrap analysis was performed based on 100 replicates [45, 46]

### Optimization of Se NPs production

Different concentrations from Na_2_SeO_3_ (1–7 mM), different ratios between cell-free bacterial metabolites and Na_2_SeO_3_ (1:1–1:11 v/v%), and different incubation times (24–96 h) were tested to determine the best conditions for the biosynthesis of Se NPs. The rate and concentration of Se NPs production were measured spectrophotometrically to elucidate the optimized parameters for the bio-formation of Se NPs.

### Characterization of the optimized biosynthesized Se NPs

Optimized Se NPs were studied and characterized using FT/IR-4000 Series Fourier transform infrared spectrometer (FT-IR, JASCO, UK), LabX XRD-6000 X-ray diffractometer (XRD, Shimadzu, Japan), JEM-2100 transmission electron microscope (TEM, JEOL, Japan), Nano-ZS90 Zetasizer (Malvern, UK), and scanning electron microscope (SEM, JEOL JSM-6510, Japan) outfitted with an EDX Genesis energy dispersive x-ray elemental analysis system (EDS).

### The anticandidal activity of the optimized Se NPs using the agar well diffusion method

The anticandidal activity of biosynthesized Se NPs was tested against *C. albicans* ATCC10231 in comparison to the standard anticandidal; miconazole using agar well diffusion method according to CLSI [40]. A 0.5 McFarland from the tested yeast was prepared and inoculated into sterile cooled molted SDA and then poured into a sterile Petri dish. Different concentrations (50 and 150 µg/mL) of optimized Se NPs and miconazole were prepared and added into 5 mm wells that were punched in the inoculated agar plates. Plates were incubated at 28 °C for 24 h and then ZOI was recorded in mm.

### Minimal inhibition concentration (MIC) and minimal fungicidal concentration (MFC)

The MIC values of the optimized Se NPs and miconazole were determined using the broth dilution method [47]. Different concentrations (0–75 µg/mL) of Se NPs and miconazole were prepared and added into sterilized Sabouraud dextrose broth (SDB) media conical flasks inoculated by 0.5 McFarland of *C. albicans* ATCC10231. Flasks were incubated at 28 °C for 24 h. The growth rate of tested yeast was measured using a Beckman DU-40 UV–Vis spectrophotometer (USA) at wavelength 600 nm against an uninoculated broth medium as a blank.

MIC flasks were inoculated into sterile cooled molted SDA medium and then poured into a sterile Petri dish. The inoculated agar plates were incubated at 28 °C for 24 h. After incubation, the growth of yeast colonies was examined, and the total was counted in colony-forming units per mL (CFU. mL^−1^) to determine MFC values [48].

### Ultra-structure study of Se NPs-treated *C. albicans* ATCC10231

Exponential-phase cultures of *C. albicans* ATCC10231 were treated by MIC value of Se NPs for 2 h at 28°C. Yeast cells were collected using centrifugation at 5000 rpm for 15 min, washed with distilled water 3 times, and then fixed using a solution with 3% glutaraldehyde. Similarly, untreated *C. albicans* ATCC10231 was prepared and used as a control. Fixed samples were dehydrated using a series of pure ethanol dilutions (10–90%), filtered using acetone, embedded in an Epon‐Araldite (1:1) mixture for 1 h, and then polymerized at 65°C for 24 h. Using copper grids, the ultrathin sections of samples were collected, stained, and observed with a TEM [49].

### Cytotoxicity effect of the biosynthesized Se NPs

The cytotoxic effect of the Se NPs was tested using normal mice keratinocyte cells. Keratinocyte cells were cultivated in a serum-free medium at the Regional Centre for Mycology and Biotechnology (Al-Azhar University, Cairo) according to the CELLnTEC protocol, Advanced Cell Systems AG (Bern, Switzerland). The experimental protocol was approved by the local ethical committee of AUHA (Al-Azhar University Housing Animals) and was conducted in compliance with the IACUC (Institutional Animal Care and Use Committee). A total of 15 × 10^3^ mouse keratinocyte cells were taken during the exponential phase of growth, planted into 96-well tissue culture plates, and left to adhere for a full day. Se NPs were then added to the appropriate wells to obtain a final concentration of 0–200 μg/mL. After incubation for 24 h, each well was filled with 20 μl of DMEM medium containing 0.4% trypan blue solution and incubated for an additional 4 h. Then, the medium was examined using the hemocytometer to count live (unstained) and dead cells (stained) under a microscope to determine the half-maximal inhibitory concentration (IC_50_) of the Se [50].

### In vitro application of the biosynthesized Se NPs as a based topical anticandidal drug

Hydrated sodium alginate (2%) was prepared in distilled water and stirred for 30 min at room temperature (25 °C). A MIC of Se NPs was added to the mixture and then stirred for 15 min to obtain alginate/Se NPs (Alg/Se). Also, the previous mixture was prepared in the presence of 1% CS to obtain Alg/CS/Se. Alg/Se and Alg/CS/Se were tested against *C. albicans* ATCC10231 using the agar well diffusion method and compared to solo Se NPs, CS, and Alg/CS.

In addition, Se NPs (MIC) was mixed with 2% panthenol cream and tested against *C. albicans* ATCC10231 compared to solo 2% panthenol cream. The increase in fold area for Se NPs mixed with panthenol or CS was determined using the equation (*B*^2^ − *A*^2^)/*A*^2^, where *A* and *B* were inhibition zones of the anticandidal agent alone (2% panthenol) and combined with Se NPs, respectively [51].

### Statistical analysis

Statistical analysis was performed on the data using SPSS version 18 software. Using One-way Analysis of Variance (ANOVA), all experiment values were presented as the mean ± standard deviation (SD). A significant threshold of *p* < 0.05 was applied [52].

## Results

### Isolation, purification, and phenotypic characterization of *bacteria* isolates

Thirteen Gram-positive, catalase-negative, non-spore-forming bacteria were recorded from the total bacterial isolates from zabady. Citric acid was positive in only 4 of them. When examined under a microscope, different morphological characteristics of bacterial cells were noted such as coccus, coccobacillus, or bacillus which are found in clusters, variable-length chains. Based on the difference in cell morphology, six LAB isolates (ESA1, ESA3, ESA5, ESA8, ESA12, and ESA13) were selected and tested for the biosynthesis of Se NPs. ESA1 and ESA12 isolates could grow at various temperatures ranging from 15 to 45 °C. At 2–6% salt, all bacterial isolates grew, except for ESA5 and ESA12 which were suppressed at 6% salt (Table 1).

**Table 1:** Biochemical tests for LAB isolates

Biochemical test	ESA1	ESA3	ESA5	ESA8	ESA12	ESA13
Growth at 15 °C	+	−	−	−	+	−
Growth at 45 °C	+	+	+	+	+	−
Growth at 2%	+	+	+	+	+	+
Growth at 6%	+	+	−	+	−	+
Catalase test	−	−	−	−	−	−
Gas from glucose	+	+	+	−	−	−
Arginine test	+	−	+	−	+	−
Arabinose	−	−	+	+	−	−
Galactose	+	+	+	+	+	+
Lactose	+	+	+	+	+	+
Maltose	+	+	+	+	+	+
Mannitol	+	−	−	−	−	−
Raffinose	+	+	+	−	+	−
Sorbitol	+	+	−	+	+	+
Nitrate reduction	−	−	−	−	−	−

### Extracellular biosynthesis of Se NPs using LAB isolates

**Fig. 1:**
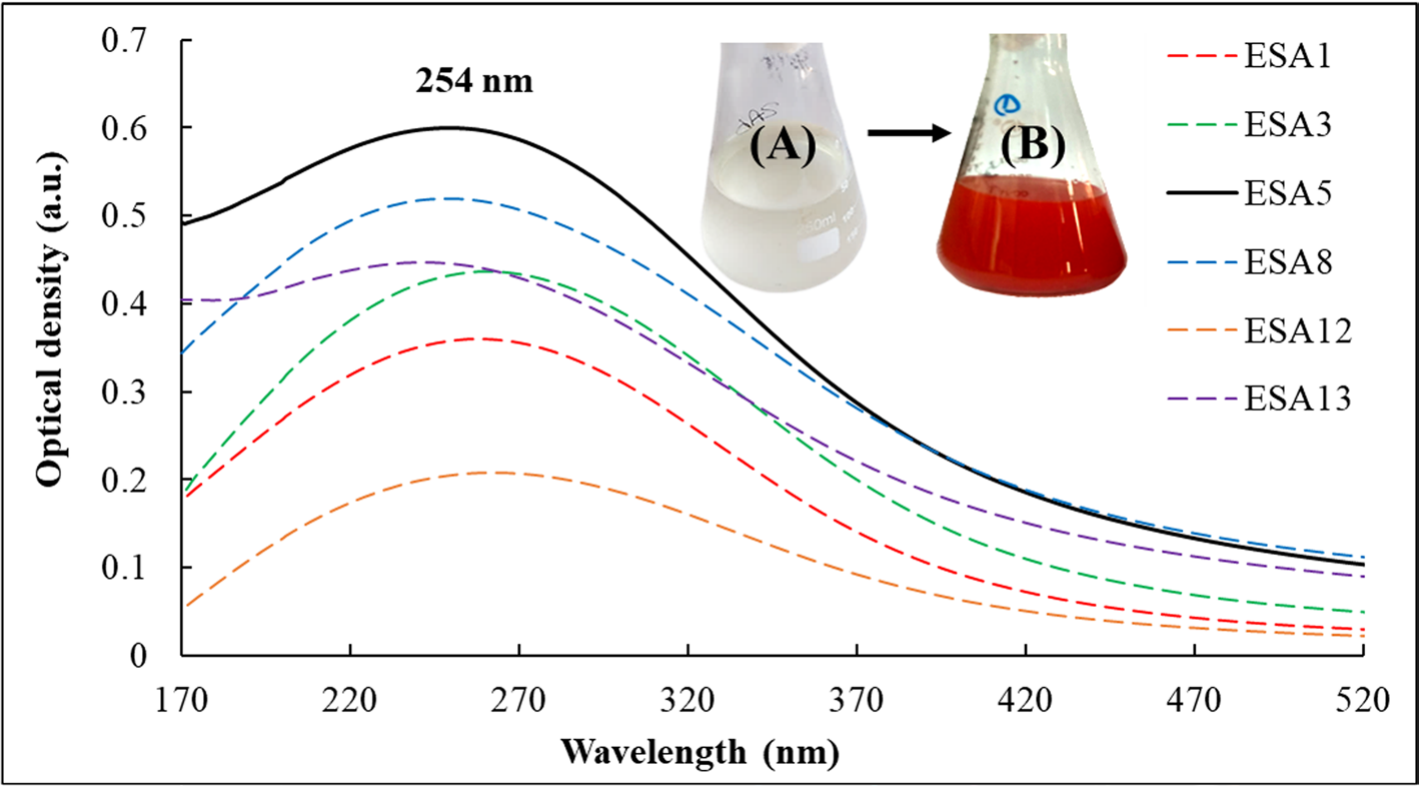
Ultraviolet–visible spectra and color change observations of Se NPs production using different LAB isolates. **A** The reaction mixture at the beginning of the reaction for ESA5 isolate. **B** The reaction mixture after incubation for 72 h

The first indication in the testing of Se NPs production by the LAB isolates is changing the medium color from pale yellow at the beginning of the experiment to red color at the end of the incubation period (Fig. 1). Among the selected isolates, ESA5 was able to produce Se NPs within 72 h at higher concentrations and faster rates than other isolates according to spectroscopy analysis results. The UV–Vis spectrum of ESA5-Se NPs showed an adsorption peak at 254 nm which matched with the characteristic properties of colloidal Se NPs [53].

### Anticandidal action of LAB-Se NPs

**Fig. 2:**
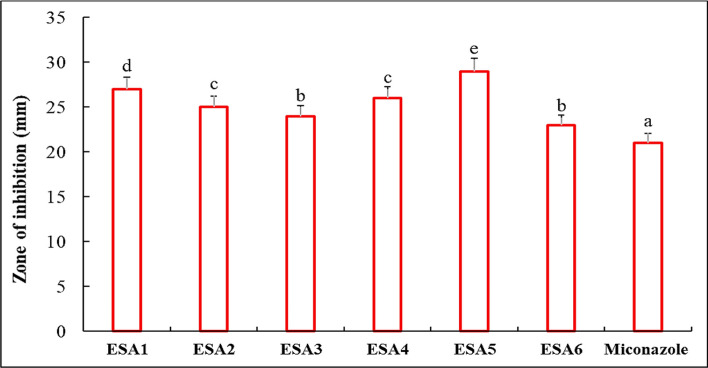
Anticandidal activity of Se NPs produced by different LAB isolates compared to miconazole

Dried Se NPs produced using different LAB isolates were tested against *C. albicans* using the agar well diffusion method compared to the standard drug, miconazole. All LAB-Se NPs revealed inhibition zones higher than miconazole confirming their potentiality as a strong anticandidal agent (Fig. 2). However, Se NPs produced by ESA5 showed the highest inhibition zone compared to other LAB-Se NPs.

### Molecular characterization of ESA5 strain

**Fig. 3:**
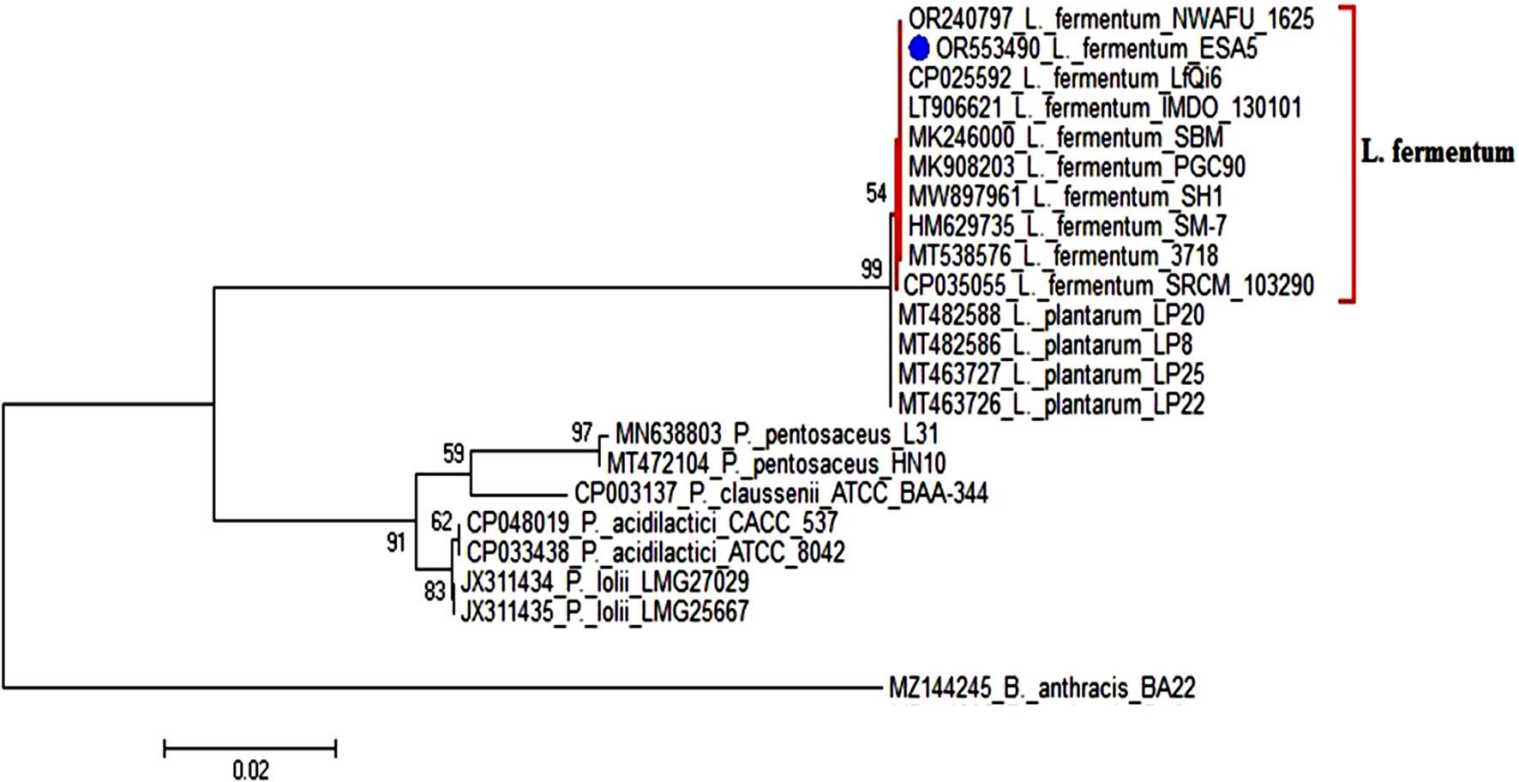
Phylogenetic tree based on 16S rRNA sequences of bacterial strain (*Limosilactobacillus* sp. ESA5). The number of branch nodes were bootstrap values (from 100 replicates) [54]. Bootstrap values above 50% are displayed. The genus *Bacillus* type strain was used as an out-group

The ESA5 isolate was chosen as a potent bio-nano-factory for Se NPs production. To verify the identification of the bacterial isolate, a phylogenetic tree was constructed using the neighbor-joining method, and the 16S rDNA sequence was analyzed. After the full 16S rRNA gene of the ESA5 isolate was sequenced, BLAST analysis revealed a relationship between the strain and the Bacillota phyla based on a comparison with sequences found in the NCBI database. The *Limosilactobacillus* genus was associated with the ESA5 strain. Strain ESA5 indeed showed 100% similarity with the strain of the type *Limosilactobacillus fermentum* (Fig. 3). The partial 16S rDNA sequence gene of *L. fermentum* ESA5 was deposited to GenBank under accession number OR553490.

### Optimization of Se NPs produced by *L. fermentum* OR553490

**Fig. 4:**
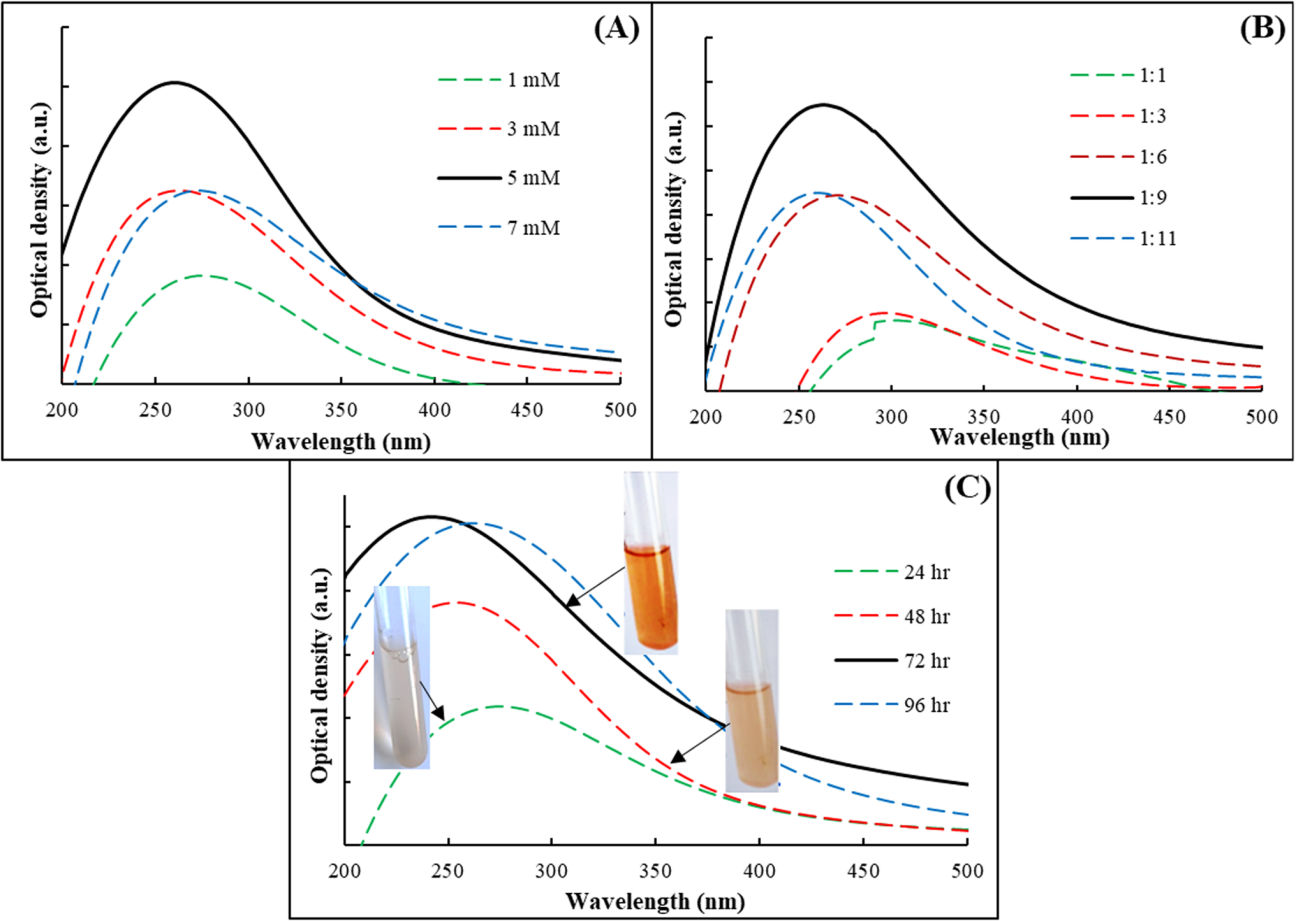
Optimization of Se NPs production. **A** Different concentrations of Na_2_SeO_3_ (mM). **B** The ratio between cell-free bacterial metabolites of *L. fermentum* and Na_2_SeO_3_ (v/v%). **C** Incubation periods (hours)

Different parameters were tested for optimal biosynthesis of Se NPs including concentrations of Na_2_SeO_3_, the ratio between cell-free bacterial metabolites and Na_2_SeO_3,_ and incubation periods (Fig. 4). The red color intensity of Se NPs production increases by increasing the concentration of Na_2_SeO_3_ from 1 to 5 mM which enhanced the biosynthesis formation of Se NPs (Fig. 4A). The mixing ratio between the cell-free bacterial metabolites and 5 mM Na_2_SeO_3_ in a 1:9 (v/v%) had the best and optimal production conditions for Se NPs formation compared to 1:11 (v/v%) (Fig. 4B). It was found that the production of Se NPs increased by increasing the incubation period (Fig. 4C).

### Characterization of the optimized biosynthesized Se NPs

**Fig. 5:**
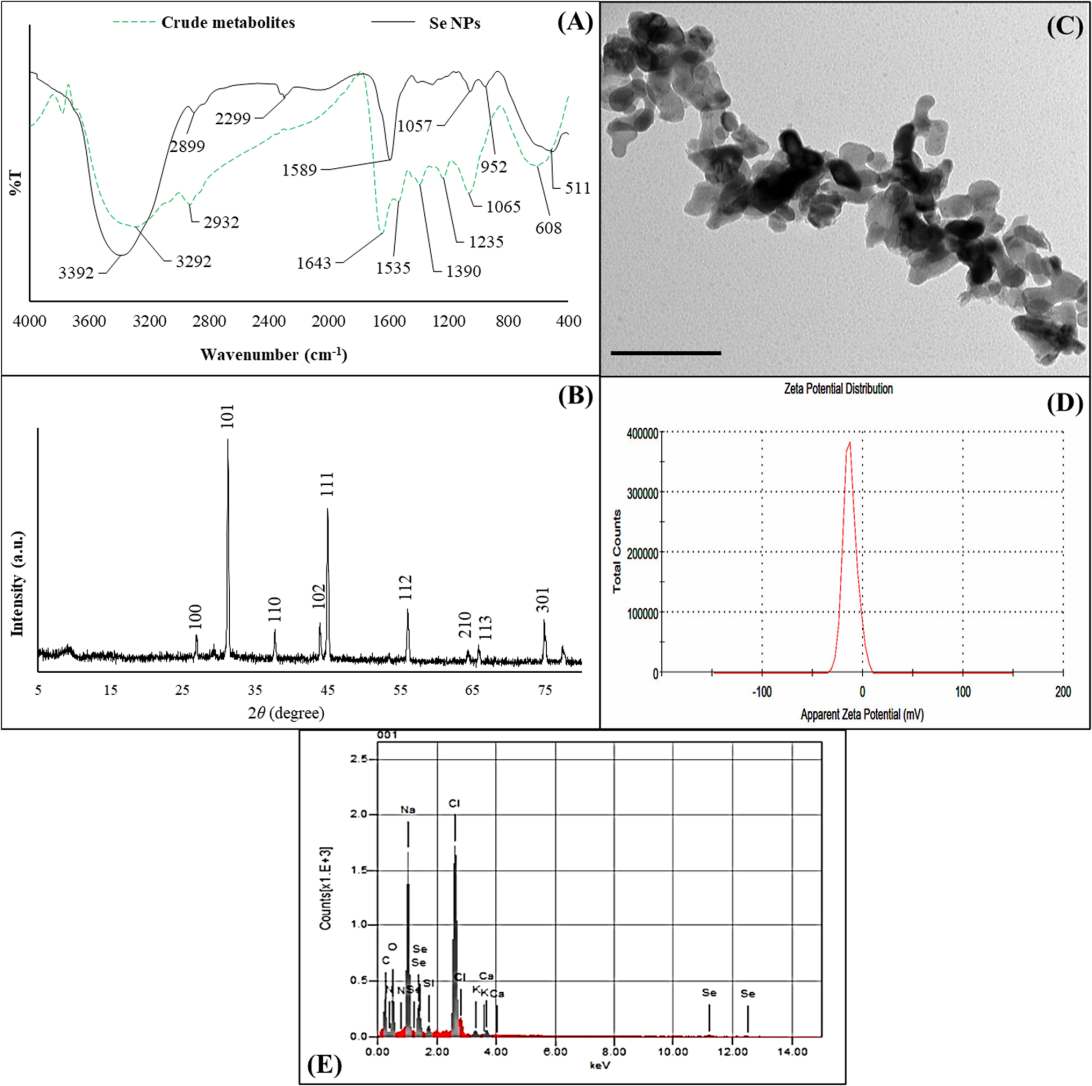
Characterization of the optimized Se NPs biosynthesized using cell-free bacterial metabolites of *L. fermentum*. **A** FT-IR. **B** XRD. **C** TEM with bar scale = 50 nm. **D** Zeta potential. **E** EDX

The FT-IR and XRD spectra were used to characterize the optimized Se NPs as well as TEM and Zeta analyses were used to study their shape, size, distribution, and potential (Fig. 5). FT-IR results of Se NPs displayed two main regions: functional group and fingerprint region (Fig. 5A). Stretching bands of symmetric (3392 cm^−1^ and 3292 cm^−1^) and asymmetric amines (2932 cm^−1^, 2899 cm^−1^ and 2299 cm^−1^) appeared at *L. fermentum* metabolites and Se NPs spectra, respectively. Aromatic and aliphatic C-N were assigned at 1643 cm^−1^, 1589 cm^−1^, 1535 cm^−1^, 1390 cm^−1^, and 1235 cm^−1^, respectively confirming the presence of proteins as stabilizing and capping agents during the biosynthesis process. Peaks at 2932 cm^−1^, 1643 cm^−1^, 1589 cm^−1^, 1535 cm^−1^, 1390 cm^−1^, and 1235 cm^−1^ in cell-free bacterial metabolites of *L. fermentum* were shifted to superior frequencies in the Se NPs pattern indicating the interaction of *L. fermentum* proteins with Se through the amine groups. The presence of NPs as a Se–O was confirmed by the metal–oxygen stretching vibrations that appeared at 1057 cm^−1^ [55]. The crystalline structure of Se NPs produced by *L. fermentum* was characterized by the XRD analysis (Fig. 5B). The results exhibited distinct characteristic peaks of Se NPs at angels 26.8°, 31.1°, 37.64°, 43.94°, 44.92°, 55.97°, 65.76°, 74.83° and 77.39° which corresponds to crystallographic planes of (100), (101), (110), (102), (111), (112), (210), (113), and (301), respectively. Lattice constants a = 4.357Å and c = 4.945Å of the obtained diffraction peaks assigned to the hexagonal structure of Se NPs which agree with the standard JCPDS data (JCPDS No. 06-0362). The absence of impurity diffraction peaks suggests that the final product was pure. Scherrer’s equation was used to evaluate the crystallite size of Se NPs: *D* = *Kλ*/(*β *cos* θ*); D: particle size, K: constant = 0.94, λ: x-ray wavelength, β: full width at half maximum and *θ*: the angle of diffraction. The crystallite size of Se NPs has been found to be ≈26 nm. TEM micrograph of Se NPs showed the successful biosynthesis of homogeneous rod-shaped NPs with average sizes ranging from 17 to 30 nm (Fig. 5C). The Zeta potential results showed the negative charge of the biosynthesized Se NPs (− 11.8 mV) as shown in Fig. 5D. The green synthetic Se NPs' energy dispersive X-ray spectrum confirms the presence of signals for nitrogen, elemental Se, sodium, oxygen, and carbon, as shown in Fig. 5E. The existence of strong signals proportional to elemental Se was exhibited by EDX spectrum. The signal of strong principal absorption, 1.37 keV was obtained for Se NPs. The peaks at 12.2, 11.2 keV, and 1.5 keV indicated the presence of elemental Se. Thus, elemental Se's existence verified the green reduction of selenium salt to elemental form. The remaining peaks were attributed to nitrogen (8.56%), carbon (33.66%), oxygen (19.81%), selenium (4.81%), and sodium (15.69%). The bioactive components, such as polysaccharides and proteins, that were attached to the surface of Se NPs were responsible for the extra peaks that followed those of nitrogen, oxygen, and carbon.

### The anticandidal activity of the optimized Se NPs

**Fig. 6:**
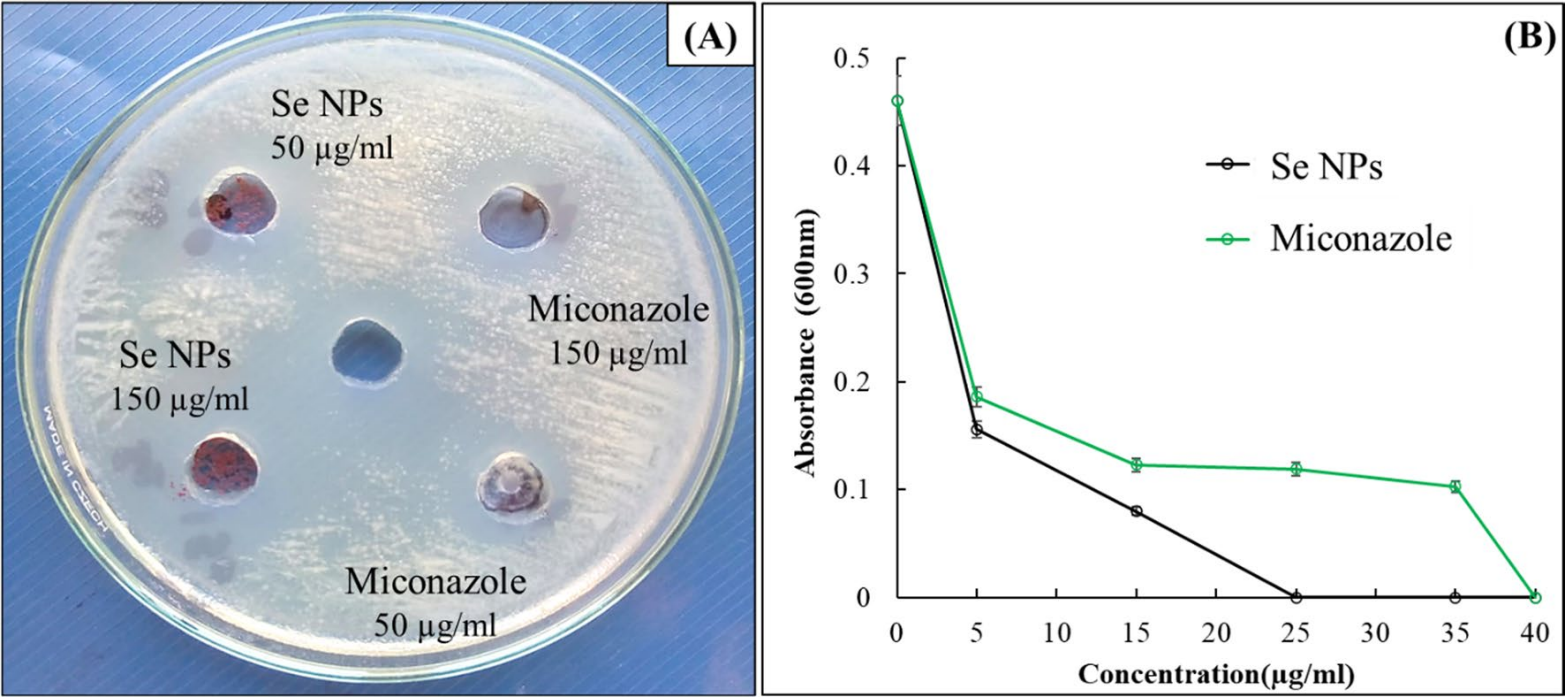
Anticandidal activity of Se NPs produced by cell-free bacterial metabolites of *L. fermentum* in comparison to miconazole using agar well diffusion method; (**A**) and minimum inhibition concentration test; (**B**)

The in vitro study showed a stronger anticandidal effect of the biosynthesized Se NPs than miconazole (Table 2 and Fig. 6). The MIC test was conducted for both Se NPs and Miconazole drug against *C. albicans* in the range of 5–40 µg/mL. The results showed that biosynthesized Se NPs had a MIC value of 25 µg/mL while the miconazole MIC value was 40 µg/mL (Fig. 6). The MFC results matched with MIC concentrations which confirmed the superior biocidal action of the biosynthesized Se NPs against *C. albicans*.

**Table 2:** Inhibition zones of *L. fermentum*-Se NPs against *C. albicans* in comparison to miconazole as a standard drug

Anticandidal agent	Concentration (µg/mL)	Inhibition zone (mean ± SD, n = 3, mm)
Se NPs	50	26 ± 0.03*
	150	31 ± 0*
Miconazole	50	18 ± 0.14*
	150	21 ± 0.06*

*Denotes significant values at *p* < 0.05

### TEM study of *C. albicans* treated by Se NPs

**Fig. 7:**
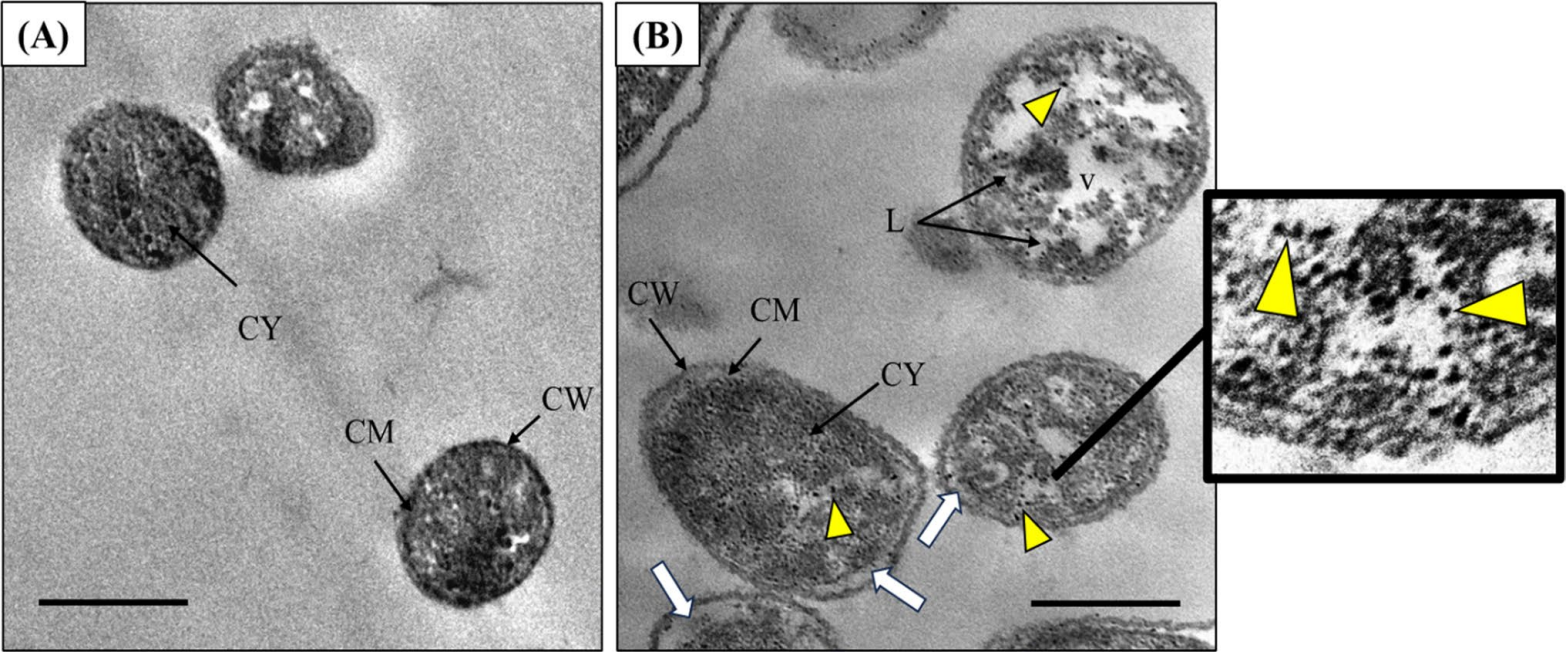
The biocidal action of Se NPs on the ultrastructure of *C. albicans* cells. **A** Untreated control cells (without NPs). **B** Treated cells with 25 µg/mL Se NPs (MIC). *CW* cell wall, *CM* cytoplasmic membrane, *CY* cytoplasm, *L* lipids formation, and *V* vacuoles. Note the visible separation between the cell and cytoplasmic membrane (white arrows) and the accumulation of NPs inside the treated cells. Bar scale = 500 nm

The anticandidal effect of Se NPs against *C. albicans* was investigated using a TEM micrograph of Se NPs-treated *C. albicans* cells compared to untreated cells (Fig. 7). The normal cells of *C. albicans* appeared to have a normal cell wall, and cell membrane, intact cytoplasm with uniformly dense and homogeneous microstructure as well as small vacuole (Fig. 7A). In contrast, Se NPs exhibit several different morphological variations in the treated *C. albicans* cells. These changes included rupture of the cell wall, wrinkling appearance of the cell wall, noticeable separation between cell and cytoplasmic membrane, and formation of a big vacuole (Fig. 7B).

### Cytotoxicity study of Se NPs

**Fig. 8:**
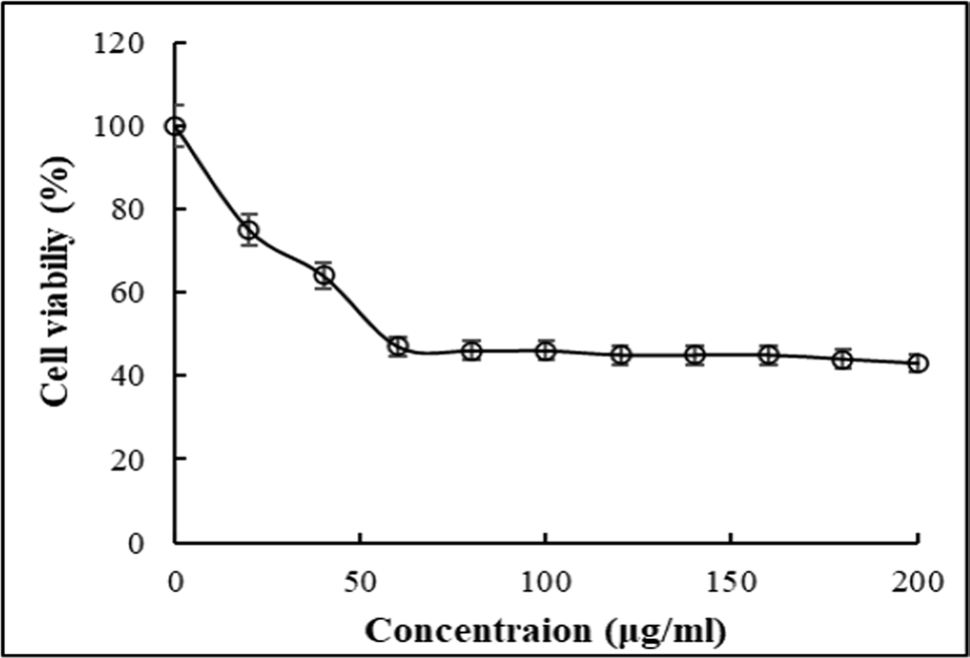
Effect of Se NPs on cell viability of the keratinocyte cells

The biosynthesized Se NPs were applied and tested against normal keratinocyte skin cells to study their toxicity (Fig. 8). Se NPs revealed an IC_50_ ≈ 41.5 ± 0.9 μg/mL. This result confirmed the safety of using lower concentrations from the biosynthesized Se NPs including MIC concentration of Se NPs (25 μg/mL).

### In vitro anticandidal potential of Se NPs as a based topical drug

**Fig. 9:**
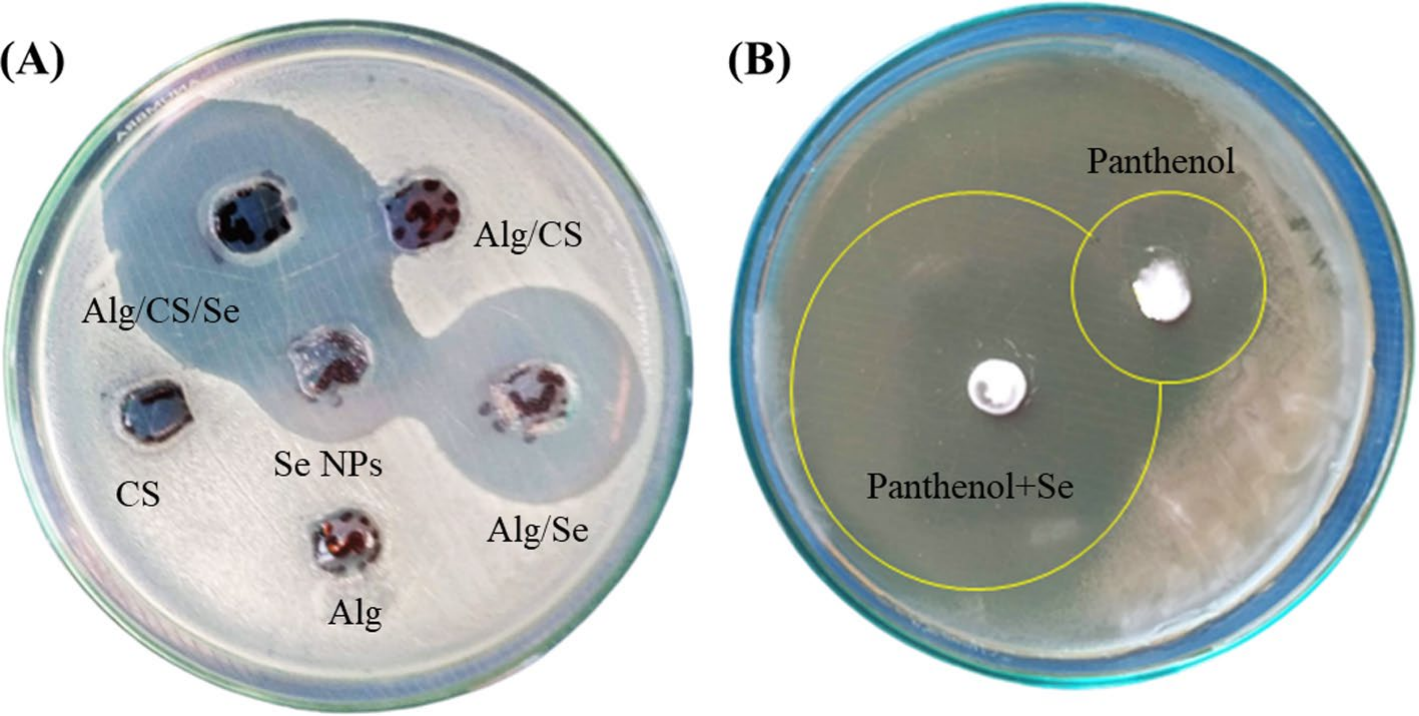
**A** Agar well diffusion test of Se NPs, Alg/Se, and Alg/CS/Se against *C. albicans*. **B** Anticandidal action of panthenol cream (2%) and panthenol cream (2%) mixed Se NPs against *C. albicans*

The anticandidal action of Se NPs, Alg/Se, and Alg/CS/Se was studied against *C. albicans* using the agar well diffusion method (Table 3 and Fig. 9A). In addition, Se NPs mixed with panthenol (2%) had a stronger anticandidal action against *C. albicans* (43 mm) than solo panthenol cream (20 mm) as shown in Fig. 9B. The biosynthesized Se NPs displayed a significant effect when companies with CS and panthenol causing an increase in the fold area reached to 9.3 and 3.6, respectively.

**Table 3:** Inhibition zones of solo and combined Se NPs against *C. albicans*

Anticandidal agent	Inhibition zone (mean ± SD, n = 3, mm)
CS	8 ± 0.14*
Se NPs	19 ± 0.03*
Alg	−ve
Alg/CS	9 ± 0.06*
Alg/Se	23 ± 0.03*
Alg/CS/Se	29 ± 0.06*

*Denotes significant values at *p* < 0.05

## Discussion

*Candida albicans* is one of the most serious fungal infections due to causing several diseases for the whole part of the body and the most dangerous problem is the resistance to antifungal agents. It can infect animals as well as humans and cause serious diseases such as candidiasis, which cause serious infection according to the infection site, cutaneous candidiasis, oral and gastrointestinal mucosal candidiasis, and vaginal canal [8]. Although there are several types of antifungal drugs such as clotrimazole, econazole, miconazole, terbinafine, fluconazole, ketoconazole, and amphotericin, were extreme irritants and might be lethal, many studies reported that azole antifungal resistance cases of *C. albicans* [56, 57]. This study aims to find an alternative green approach to overcome candidal infection problems especially *C. albicans* resistance to current commercial drugs.

Nanomaterials have piqued the interest of scientists due to their applications in chemistry, medicine, and other sciences. Among nanomaterials, nanometals of selenium, silver, copper, and gold display novel chemical, physical, and biological characteristics that were used as strong anticandidal agents [58–61]. Recently, biological methods for NPs production have made tremendous progress due to their low cost, simplicity, and quick procedure. Selenite reduction by microorganisms has received considerable attention among the many forms of selenium due to its toxicity [62]. In the current work, Se NPs were extracellularly biosynthesized using the cell-free supernatant of *L. fermentum* OR553490 as a safe, cheap, and simple one-step. Classical, biochemical, and molecular techniques have been used to confirm the identification of the *L. fermentum* strain at the species level. The formation of red color by the cell-free supernatant of *L. fermentum* implies that this bacterium's crude metabolites may bio-reduce poisonous and colorless selenite to nontoxic and red metallic Se NPs. The production of a distinctive red color was caused by the excitation of surface plasmon vibrations of Se [63]. Even so, the exact approach by which bacteria produce Se NPs has not yet been recognized. Previous investigations demonstrated that NADH and the NADH-dependent nitrate reductase enzyme play essential roles in the forming of metal nanoparticles. Dwivedi et al. [64] suggested that NADH and NADH-dependent reductases as redox agents are responsible for the reduction of metal ions of selenite to selenium nanospheres using the culture supernatant of *Pseudomonas aeruginosa*. Reductases can also act as capping agents, ensuring the production of thermodynamically stable nanostructures [65]. *L. fermentum* was reported to produce NADH and NADH-dependent reductase enzymes [66–69]. As a result, it is anticipated that a multi-component redox system in the cell-free supernatant of *L. fermentum*, comprising NADH, and most likely NADH-dependent reductases, would function to catalyze the biosynthesis of Se NPs.

The biosynthesis of Se NPs was optimized by testing different parameters such as concentrations of Na_2_SeO_3_, the ratio between cell-free bacterial metabolites and Na_2_SeO_3,_ and incubation periods. It was recorded that mixing of mixing of cell-free bacterial metabolites with 5 mM Na_2_SeO_3_ in a 1:9 (v/v%) for 72 h were the best conditions for the biosynthesis of Se NPs whereas higher mixing ratio (1:11 v/v%) did not favorable for the Se NPs formation due to the excess amount of reducing agents [70, 71]. El-Dein et al. [72] recorded that the mixing ratio of 1:3 v/v% enhanced the production of NPs using bacterial metabolites. However, the red color intensity increased by the incubation time which refers to the high reduction rate, *L. fermentum* started to change the yellowish media color after the first 12 h and gave a high yield of Se NPs after 72 h. The small amount production of Se NPs at lower concentrations of Na_2_SeO_3_ solutions (1, 3 mM) might be due to the low amounts of enzymes' substrate, while higher concentration of Na_2_SeO_3_ solution (7 mM) did not enhance the Se NPs formation due to its toxicity [65, 73]. El-Saadony et al. [64] obtained different results in the optimization condition for the probiotic bacteria *L. paracasei*. The best concentration of Na_2_SeO_3_ was 4 mM and the reaction mixture was incubated for 32 h. The ratio between Na_2_SeO_3_ and the cell-free supernatant of *L. fermentum* was increasable relation, increasing the salt concentration with the cell-free supernatant of *L. fermentum* that is rich with reducing enzymes led to increasing the Se NPs production.

The biosynthesis of Se NPs was confirmed by the presence of an absorbance peak at ≈254 nm. Several studies reported different absorbance peaks for Se NPs for example, Hemalatha et al. [73] studied Se NPs which have an absorbance peak of 290 nm while El-Saadony et al. [74] reported other results at 263 nm and 300 nm, respectively. The absorbance peak changes towards longer wavelengths as particle size increases [53]. Only when the particle size is 100 nm or larger, Se NPs display a consistent absorption peak in the wavelength band above 300 nm. The absorption peak typically changes towards red, and the peak intensity diminishes as nanoparticle size increases [75].

There are common problems related to the stability of NPs. Agglomeration and aggregation of NPs are some of the biggest problems that decrease their use in different applications. Capping agents have a noticeable role in preventing NPs aggregation [76]. The FT-IR analysis confirmed the presence of proteins as capping agents that surrounded the NPs. Some studies confirm that the binding of protein capping agents increases the stability of NPs through cysteine and amine residues, which prevent the accumulation and aggregation of NPs [77]. Also, the amide III band of proteins was documented during the use of *L. acidophilus* in the biosynthesis of Se NPs [18]. This capping agent may have negative or positive charges that also have a relative role in the stability of NPs and important characteristics of the colloidal dispersion of NPs. Se NPs-*L. fermentum* was found to have a negative charge of -11.8mV which causes a repulsion force between NPs grains and increases their stability. Laslo et al. [34] reported that *L. casei* produced Se NPs with a maximum value of Zeta potential of – 23 mV. It is worth noting that the zeta potential surface charge of NPs also has a great role in antimicrobial activity through the electrostatic adhesion interaction between NPs and microbial cell membranes [78]. Different bacteria such as *L. lactis*, *Lactobacillus* sp., and *Bifidobacter* sp. were used in Se NPs production as a green nano-factory, however, one of their disadvantages was the large nano-sized particles which ranged from 100 nm-550 nm [33]. In contrast, *L. fermentum* produced Se NPs with a size range of 17–30 nm. The XRD results confirmed purity and crystalline nature of the produced Se NPs. The XRD patterns of the *L. fermentum*-Se NPs matched with *L. paracasei*-Se NPs which showed eight peaks at 2*θ* of 28.61°, 31.19°, 40.01°, 45.02°, 56.21°, 66.23°, 75.11°, and 84.74° which corresponds to crystallographic planes of 100, 101, 110, 102, 111, 201, 112 and 202 [64].

The biosynthesized Se NPs were reported by several studies as a strong antimicrobial agent [53, 77, 79, 80]. Similarly, the biosynthesized Se NPs using *L. fermentum* had stronger biocidal action against *C. albicans* with MIC and MFC values of 25 µg/mL compared to the standard drug miconazole (40 µg/mL). El-Saadony et al. [64] reported a MIC and MFC of 55 and 80 μg/mL, respectively of LAB-Se NPs against *C. albicans*. Shakibaie et al. [81] biosynthesized Se NPs by using *Bacillus* species Msh1 and had MIC of 70 μg/mL against *C. albicans*. Besides the low MIC value of the biosynthesized Se NPs, the cytotoxic effect was performed on the normal keratinocyte cells by the trypan blue method. A low toxic effect of Se NPs with IC_50_ value ≈ 41.5 μg/mL.

Nevertheless, the exact biocidal mode of action of NPs has not been determined, several studies recommended that selenium can increase the ROS formation. Small-sized reduced selenium ions can infuse easily through the cell wall of the fungus, attach, and affect negatively the respiratory sequence and ATP [82]. In addition, selenium has a denaturation action by increasing the superoxide radical levels and causes an increase the fungal cell death [83]. TEM micrographs of treated *C. albicans* by Se NPs showed the accumulation of Se NPs inside the treated cells in the cytoplasmic membrane which might cause the interactions between the microbial cellular components and NPs. This treatment led to numerous modifications in the treated cells compared to normal cells. These changes involved morphological abnormalities in the cell wall and cytoplasmic membrane, decreasing cytoplasmic and DNA content, large vacuoles, and lipids formation.

The current study also investigated the potent anticandidal potential of Se NPs in combination with CS and panthenol. Both CS and panthenol were recommended and used as anticandidal agents against different pathogenic microbes [16, 84]. However, many *Candida* species recently showed several resistant mechanisms against these antimicrobial agents include active efflux systems and decrease the antimicrobial agent accumulation inside their cells [85, 86]. Alg was reported to enhance the antifungal activity against candidal biofilms [87]. Alg was also used to reduce the toxicological effects of NPs and to expand its clinical uses in nanocarriers and drug delivery systems [88]. Kalińska et al. [89] in vitro study displayed that the combination of NPs such silver and copper NPs with cosmetics such as panthenol, vitamin C, sodium lactate, and marigold flower extract act as strong antimicrobial agents compared to their solo components. When CS and panthenol were combined with Se NPs, there was a strong anticandidal effect against *C. albicans* with an increase in fold area of 9.3 and 3.6, respectively. These results increase the possibilities of using the biosynthesized Se NPs in different medical and industrial applications as promising solo or combined anticandidal agents.

## Conclusion

The current study highlighted a novel treatment for dangerous candidal infections using Se NPs. A cell-free supernatant of *L. fermentum* (OR553490) was used in the extracellular biosynthesis of Se NPs. 5 mM Na_2_SeO_3_ and 1:9 (v/v%) mixing ratio between Na_2_SeO_3_ and *L. fermentum* metabolite enhanced the bio-production of Se NPs for 72 h. The biosynthesized Se NPs using *L. fermentum* revealed stronger anticandidal action against *C. albicans* compared to Se NPs synthesized by other LABs. They mark their effectiveness as anticandidal at lower concentrations, moreover, having a low toxic effect on normal cells. The obtained results recommended the combination between *L. fermentum* Se NPs with CS and/or panthenol as developed potential anticandidal agents to compete the *C. albicans* resistance problems. Furthermore, the toxicity and anticandidal action of Se NPs needs to be studied in vivo with an animal model.

## Data Availability

The partial sequence of the 16S ribosomal RNA gene of *Limosilactobacillus fermentum* strain ESA5 obtained in the current study was deposited in the NCBI GenBank database under accession number: OR553490 and is available at the following URL: https://www.ncbi.nlm.nih.gov/nuccore/OR553490. The datasets generated during the current study are available from the corresponding author upon reasonable request.

## Abbreviations

Se NPs: Selenium nanoparticles
CS: Chitosan
Alg: Alginate
UV–Vis: Ultraviolet–visible
FT-IR: Fourier transform-infrared spectroscopy
XRD: The X-ray diffraction
TEM: Transmission electron microscopy
EDS: Elemental analysis system
IC_50_: Half maximal inhibitory concentration
ANOVA: One-way analysis of variance
